# *MET* Gene Copy Number Alterations and Expression of MET and Hepatocyte Growth Factor Are Potential Biomarkers in Angiosarcomas and Undifferentiated Pleomorphic Sarcomas

**DOI:** 10.1371/journal.pone.0120079

**Published:** 2015-04-06

**Authors:** Katja Schmitz, Hartmut Koeppen, Elke Binot, Jana Fassunke, Helen Künstlinger, Michaela A. Ihle, Carina Heydt, Eva Wardelmann, Reinhard Büttner, Sabine Merkelbach-Bruse, Josef Rüschoff, Hans-Ulrich Schildhaus

**Affiliations:** 1 Institute of Pathology, University Hospital Cologne, Cologne, Germany; 2 Institute of Pathology, University Hospital Göttingen, Göttingen, Germany; 3 Genentech Inc., South San Francisco, California, United States of America; 4 Gerhard Domagk Institute of Pathology, University Hospital Münster, Münster, Germany; 5 Targos Molecular Pathology GmbH, Kassel, Germany; Johns Hopkins University, UNITED STATES

## Abstract

Soft tissue sarcomas are a heterogeneous group of tumors with many different subtypes. In 2014 an estimated 12,020 newly diagnosed cases and 4,740 soft tissue sarcoma related deaths can be expected in the United States. Many soft tissue sarcomas are associated with poor prognosis and therapeutic options are often limited. The evolution of precision medicine has not yet fully reached the clinical treatment of sarcomas since therapeutically tractable genetic changes have not been comprehensively studied so far. We analyzed a total of 484 adult-type malignant mesenchymal tumors by *MET* fluorescence *in situ* hybridization and MET and hepatocyte growth factor immunohistochemistry. Eleven different entities were included, among them the most common and clinically relevant subtypes and tumors with specific translocations or complex genetic changes. MET protein expression was observed in 2.6% of the cases, all of which were either undifferentiated pleomorphic sarcomas or angiosarcomas, showing positivity rates of 14% and 17%, respectively. 6% of the tumors showed hepatocyte growth factor overexpression, mainly seen in undifferentiated pleomorphic sarcomas and angiosarcomas, but also in clear cell sarcomas, malignant peripheral nerve sheath tumors, leiomyosarcomas and gastrointestinal stromal tumors. MET and hepatocyte growth factor overexpression were significantly correlated and may suggest an autocrine activation in these tumors. *MET* FISH amplification and copy number gain were present in 4% of the tumors (15/413). Two samples, both undifferentiated pleomorphic sarcomas, fulfilled the criteria for high level amplification of *MET*, one undifferentiated pleomorphic sarcoma reached an intermediate level copy number gain, and 12 samples of different subtypes were categorized as low level copy number gains for *MET*. Our findings indicate that angiosarcomas and undifferentiated pleomorphic sarcomas rather than other frequent adult-type sarcomas should be enrolled in screening programs for clinical trials with MET inhibitors. The screening methods should include both *in situ* hybridization and immunohistochemistry.

## Introduction

Soft tissue sarcomas represent a heterogeneous tumor group which consists of over 50 different histologic subtypes [1]. Taken together estimated 12,020 new cases will be diagnosed and 4,740 soft tissue sarcomas related deaths can be expected in the United States in 2014 [2]. Soft tissue sarcomas are often diagnosed at advanced stage, because symptoms may be absent for a long time. At time of diagnosis, 10–25% of patients present hematogenous metastases, mostly to lung, bone and liver [3]. Standard treatment for localized tumors is surgery. High-grade tumors are in certain clinical settings treated with radiation therapy, and also conventional chemotherapy is beneficial in some patients. In advanced stage, chemotherapy is currently used as standard treatment [4]. However, these treatments often have adverse effects or are ineffective, e.g., for retroperitoneal sarcomas the 5-year local control rate is only 40–71% and the 5-year survival rate is low with 51–60% [5]. Sarcomas of the extremities have a high local recurrence rate of 30–50% and half the patients die from their disease [6].

For some sarcoma subtypes tumor specific molecular aberrations such as gene translocations, amplifications or mutations have been identified, however, their exploitation as therapeutic targets has been limited. The best example for molecular targeted therapy in mesenchymal tumors is treatment with tyrosine kinase inhibitors in gastrointestinal stromal tumors (GIST) which carry activating *KIT* or *PDGFRA (platelet derived growth factor receptor alpha)* mutations in approximately 90%. For patients with other soft tissue sarcoma entities, however, new effective and reliable molecular based treatments are desirable. Targeting tyrosine kinases has given promising results in many malignancies, but comprehensive studies on soft tissue sarcomas are still lacking.

One of those therapeutically tractable tyrosine kinases is MET which is currently subject to many clinical trials with promising results. MET is a transmembrane tyrosine kinase receptor and is also known as hepatocyte growth factor receptor (HGFR) [7]. It is mainly located at the surface of epithelial cells [8] and has been found in fibroblasts [9], endothelial cells [10], pericytes and smooth muscle cells [11]. It is activated in a paracrine manner by its only known ligand HGF/SF (hepatocyte growth factor or scatter factor) which is secreted by mesenchymal cells [9]. The HGF/MET pathway promotes cell proliferation, motility and angiogenesis. Physiologically it is activated during embryogenesis, morphogenesis, tissue regeneration and repair. Improper activation may lead to tumorigenesis, tumor angiogenesis, invasion and metastasis. Aberrant activation may be ligand driven by paracrine and autocrine mechanisms or based on ligand independent mechanisms such as receptor overexpression, activating gene mutations, gene amplification or altered transcription [12, 13]. MET seems to play a role in the pathogenesis of a variety of tumors such as lung, liver, renal and gastric cancer [14–20]. Some soft tissue sarcomas have also been reported to overexpress MET, among them synovial sarcomas, leiomyosarcomas, rhabdomyosarcomas, fibrosarcomas and other types [21–27]. An overexpression of the MET ligand HGF could be found—among others—in malignant pleural mesotheliomas, gastric carcinomas, gliomas as well as in some soft tissue sarcomas [22, 24].

The HGF/MET pathway can therapeutically be targeted in a variety of ways, e.g., blockade of the ligand-receptor interaction using HGF antagonists, HGF neutralizing antibodies or MET antibodies, inhibition of receptor dimerization and inhibition of kinase activity with interruption of downstream signaling using small molecule inhibitors [28]. There are currently various HGF/MET inhibiting drugs in clinical trials. Most patients included in those studies are treated for carcinomas and only rarely for sarcomas. Among all clinical trials listed at clinicaltrials.gov [29] for MET and HGF inhibitors four studies are open explicitly for patients with sarcomas and there are multiple studies open to patients with not further specified solid tumors, which could include also soft tissue sarcomas (June 2014). Few sarcoma subgroups are already enrolled in clinical trials for ARQ 197 (Tivantinib) and PF-02341066 (Crizotinib) [29]. Only few preclinical studies, however, dealt with inhibition of HGF/MET axis in sarcomas, but they could show antitumoral effects on growth, viability, invasion and metastasis *in vitro* and *in vivo*. Analyzed cell lines included clear cell sarcomas [22], rhabdomyosarcomas [30], osteosarcomas [31], alveolar soft part sarcomas [23], as well as cell lines and murine models of leiomyosarcomas [32] and malignant peripheral nerve sheath tumors (MPNST) [33]. However, a systematic and comprehensive prevalence study of MET and HGF alterations in soft tissue sarcomas has not yet been published.

In this study we aimed at investigating the HGF/MET axis in a comprehensive and well characterized cohort of various adult-type soft tissue sarcomas and GIST. Our goal was to determine the prevalence of the three major mechanisms of MET activation, i.e., *MET* gene amplification, and MET as well as HGF protein overexpression in clinically relevant sarcoma entities. We describe frequencies and patterns of *MET* gene copy number alterations by fluorescence *in situ* hybridization (FISH) as well as MET and HGF expression by immunohistochemistry. Our results were obtained by up-to-date biomarker assays and provide first comprehensive epidemiologic data for ongoing and upcoming clinical trials with MET/HGF inhibitors in soft tissue sarcomas and GIST.

## Materials and Methods

### Tumor samples

A total of 484 malignant mesenchymal tumors were selected for analysis from the files of the GIST and sarcoma registry Cologne/Bonn, Germany. The samples were collected between 1992 and 2011. All tumors were reviewed by experienced pathologists (HUS, EW or RB) based on histology, immunohistochemistry and molecular findings according to the current WHO classification [1]. Eleven different mesenchymal tumor entities with high clinical relevance were selected for this study: atypical lipomatous tumors/well differentiated liposarcomas (ALT; n = 43), dedifferentiated liposarcomas (DDLS; n = 70), myxoid liposarcomas (MLS; n = 30), pleomorphic liposarcomas (PLS; n = 10), leiomyosarcomas (LMS; n = 69), angiosarcomas (ASA; n = 41), synovial sarcomas (SS; n = 15), malignant peripheral nerve sheath tumors (MPNST; n = 24), undifferentiated pleomorphic high grade sarcomas (UPS; n = 36), clear cell sarcomas (CCS; n = 13), and gastrointestinal stromal tumors (GIST; n = 133). These entities were selected i) to include the most frequent sarcoma subtypes (e.g., ALT, DDLS and LMS), ii) to compare tumors with known underlying genetic alterations (translocation positive sarcomas, e.g., MLS or SS; sarcomas with gene amplifications, e.g., ALT or DDLS; tumors with activating mutations, i.e., GIST) with neoplasms which are characterized by complex genetic changes (e.g., UPS, MPNST), and iii) to include as well entities for which previous data indicate that MET alterations might play a role (e.g., clear cell sarcomas). Formalin-fixed, paraffin-embedded tumor tissue was used to construct tissue microarrays (TMA). Each case was represented by two cores, measuring 1mm in diameter (from two representative tumor areas). The clear cell sarcomas and some of the angiosarcomas (12/41) were analyzed on conventional tissue slides. A subset of the undifferentiated pleomorphic sarcomas initially analyzed on the TMA was analyzed on conventional tissue slides subsequently to all other experiments and statistic calculations to assess heterogeneity of *MET* gene copy numbers.

This study was conducted in accordance to the Declaration of Helsinki and to guidelines of the local Ethics Committees. Cases were derived from our consultation files and originated from various primary sites. Samples were obtained as part of routine clinical care with verbal informed consent from each patient. Patients and their physicians agreed in sending paraffin blocks to us for further analyses. Met studies within this work were carried out anonymously as a retrospective epidemiologic investigation. Therefore, the Ethics Committee of the University Hospital Cologne, Germany, waived the need for a specific approval or written informed consent.

### Immunohistochemistry

For immunohistochemical determination of protein expression antibodies against MET (CONFIRM anti-Total c-MET (SP44), Rabbit Monoclonal Primary Antibody, Ventana, Tucson, AZ) [34] and HGF (HGF-α, polyclonal rabbit, dilution 1:50, Zytomed, Berlin, Germany, catalog number: A01-8135) were used. HGF immunohistochemistry was performed by a peroxidase-conjugated avidin–biotin method using an automatic stainer (medac Diagnostika, Wedel, Germany). 4μm thick sections of paraffin-embedded tumor tissue were mounted on positively charged, adhesive glass slides and dried at 37°C overnight. The sections were deparaffinized in xylene, rehydrated in ethanol and washed with buffer (Tris buffered saline & Tween 20). The tissue was pretreated with citrate buffer (pH 6.0) in a microwave oven. Sections were incubated with the primary antibody for 30min at room temperature. Hydrogen-peroxide was incubated for 5min and washed off with buffer. After enhancer incubation and a washing step with buffer, the sections were incubated for 15min with the polymer Poly-HRP-GAM/R/RIgG followed by incubation for 8min with 3,3’-diaminobenzidine (DAB) and washed with buffer. Sections were counter stained with hematoxylin, washed, dehydrated with ethanol and coverslipped. IHC for MET was performed as previously described [34]. The staining reactions for both antibodies were graded on a four-tiered system principally based on the scoring system used by Koeppen et al. for validation of this antibody in lung cancer but slightly modified for the use in tissue microarrays [34]: 0, no staining or <10% of tumor tissue stained at any intensity; 1+, weak staining in ≥10%; 2+, moderate staining in ≥10% of tumor cells; 3+, strong staining in ≥10% of tumor cells. Strong staining intensity was defined by the level of MET and HGF expression in the H441 and EBC-1 cell lines and in breast carcinoma tissue, respectively. Only membranous and cytoplasmic staining reactions were evaluated, while nuclear staining was neglected if occurred. Cases with 0 or 1+ staining were considered as negative, 2+ and 3+ staining as positive.

### Fluorescence *in situ* hybridization

The fluorescence *in situ* hybridization (FISH) for analysis of *MET* gene copy numbers was performed with the ZytoLight SPEC *MET*/CEN7 Dual Color Probe (ZytoVision, Bremerhaven, Germany). Hybridization was carried out as previously published [35]. Briefly, 4μm thick sections were cut, pretreated and 5–10μl *MET*/CEN7 probe was applied on the tumor tissue. Denaturation, 75°C for 10 minutes, and hybridization, 37°C overnight, were carried out in the ThermoBrite humidity chamber (Abbott Molecular, Wiesbaden, Germany). After posthybridization washes the slides were counterstained with DAPI and coverslipped. Using a fluorescence microscope (Leica, DM 5500, Wetzlar, Germany) equipped with the appropriate filters, the whole tumor area was evaluated with a 63x or 100x objective. The numbers of *MET* and centromere 7 signals were counted in 60 nuclei obtained from three different areas with the highest gene count. Average gene copy number and ratio (*MET*/CEN7) as well as the percentages of tumor cells with ≥4, 5 and 15 gene copies were calculated for each tumor. Tumors were sorted into four categories based on a *MET* scoring system previously introduced by us in a lung cancer study [36]. In brief, the four categories were as follows:

1. **high-level amplification** was defined as
  - *MET*/CEN7 ratio ≥2.0 or
  - average *MET* gene copy number per cell of ≥6.0 or
  - ≥10% of tumor cells containing ≥15 *MET* signals.
2. **intermediate-level copy number gain** being defined as
  - ≥50% of cells containing ≥5 *MET* signals and
  - criteria for high-level amplification are not fulfilled
3. **low-level copy number gain** was defined as
  - ≥40% of tumor cells showing ≥4 *MET* signals and
  - criteria for high-level amplification or intermediate-level copy number gain are not fulfilled
4. all other tumors were classified as **negative**.

FISH positivity was defined by categories i-iii, including amplification and copy number gains.

### Statistical analysis

For statistical analyses SPSS 21.0 software (IBM, Armonk, NY, USA) was used. All tests were two-sided, with a 95% confidence interval.

## Results

### Protein expression

Immunohistochemical staining results were available for MET in 388/484 and for HGF in 466/484 tumors. Results for both parameters were obtained in 382 tumors.

**Fig 1:**
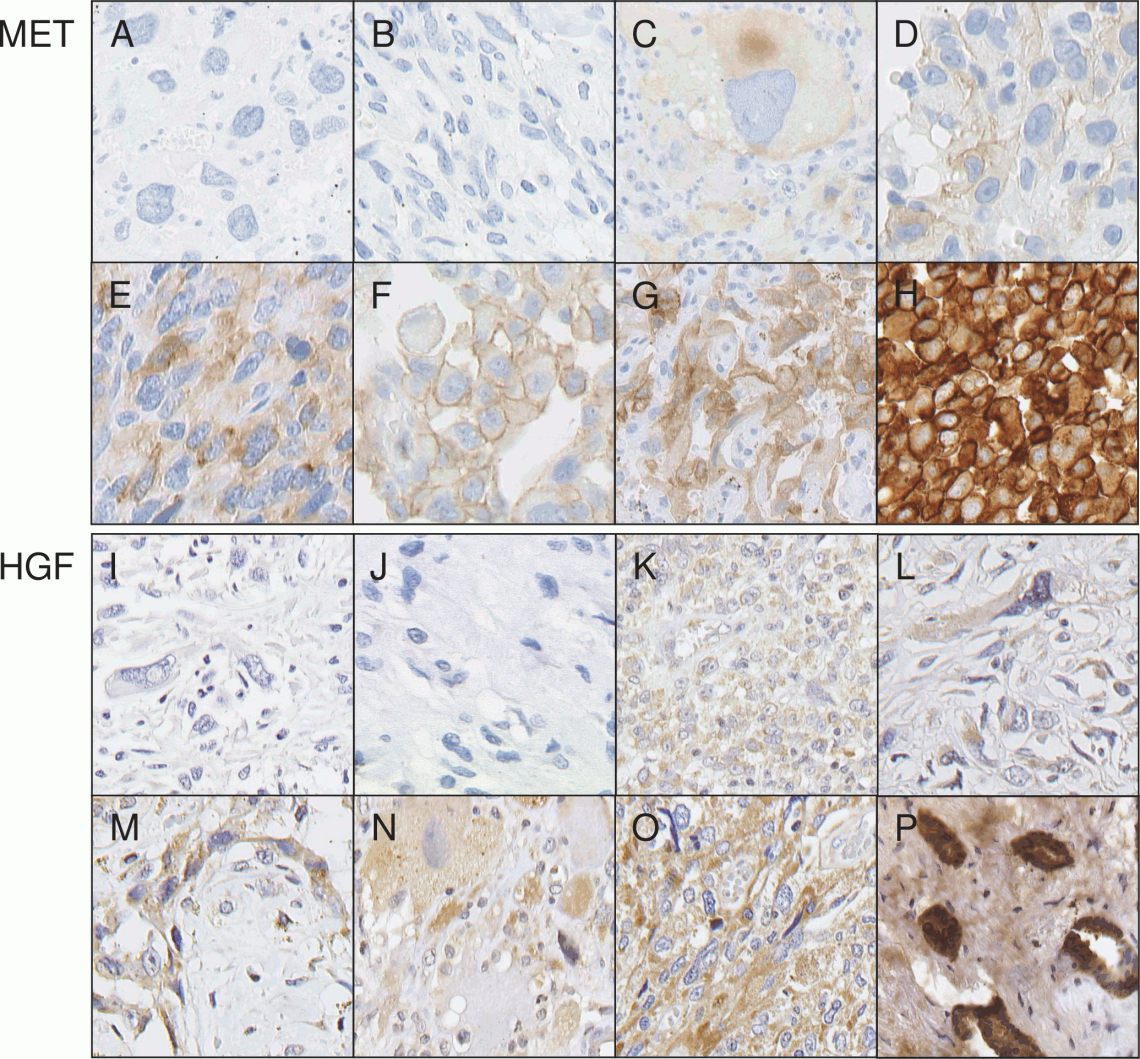
MET and HGF expressions. Representative sarcomas showing range and pattern of immunohistochemical staining for MET (A-H) and HGF (I-P). (A, B, I, J) staining intensity score = 0, (C, D, K, L) score = 1+, (E, F, G, M, N, O,) score = 2+, (H, P) score = 3+. (A, C, E, N) undifferentiated pleomorphic sarcomas, (B, D, F, G, K, M) angiosarcomas, (H) control cell line, (I, L, O) leiomyosarcomas, (J) dedifferentiated liposarcoma, (P) control tissue breast cancer.

*MET*: Out of 388 analyzable samples 10 tumors (2.6%) were MET immunopositive (Table 1; Fig. 1). Notably, all tumors with MET overexpression were either angiosarcomas or undifferentiated pleomorphic sarcomas. Angiosarcomas were positive in 5/29 tumors (17%) and undifferentiated pleomorphic sarcomas in 5/36 tumors (14%). All positive cases had moderate staining intensity in ≥10% of tumor cells (IHC score 2+). None of the cases showed strong staining (score 3+). Among the 378 MET negative cases weak staining (score 1+) was observed in 20 tumors (in DDLS (3/70), LMS (2/69), ASA (5/29), SS (1/14), MPNST (3/24) and UPS (6/36)). ALT, MLS, PLS and GIST did not show any staining in any of the cases. Correlation between entity and MET immunoscore was statistically significant (p<0.001, Chi Square). All MET positive tumors, i.e., angiosarcomas and undifferentiated pleomorphic sarcomas, were characterized by complex karyotypes and none of the tumors with specific chromosomal translocations or mutations was positive (p = 0.003, Chi Square, p = 0.002 Fisher’s exact). MET immunoscore (score 0–2) correlated significantly with FNCLCC (French cancer society, i.e., Fédération Nationale des Centres de Lutte Contre le Cancer) histological grading for sarcomas (p = 0.016, Chi Square): for 276 tumors both MET score and FNCLCC grading were available which included 8 MET positive tumors. Seven of them were FNCLCC grade 3 and one was FNCLCC grade 2. Higher grade was associated with stronger MET expression since 4% of grade 3, 2% of grade 2 and none of grade 1 tumors were MET positive. In the group of angiosarcomas there was no significant correlation between MET immunoscore and particular pathogenesis (sporadic vs. radiation induced; p = 0.583), or with *MYC* amplification status (p = 0.662). For a subset of angiosarcomas and for clear cell sarcomas we could not obtain valid data on MET immunohistochemistry (Table 1). This is due to limited tissue resources.

**Table 1:** Immunohistochemical expression scores of MET and HGF.

Tumor type		MET immunoscore*				HGF immunoscore*			
		(n; % of evaluable cases)				(n; % of evaluable cases)			
	n	negative	weak	moderate	n.a.	negative	weak	moderate	n.a.
		0	1+	2+		0	1+	2+	
ALT	43	42 (100)	0	0	1	42 (100)	0	0	1
DDLS	70	65 (96)	3 (4)	0	2	61 (87)	9 (13)	0	0
MLS	30	21 (100)	0	0	9	22 (100)	0	0	8
PLS	10	10 (100)	0	0	0	10 (100)	0	0	0
LMS	69	67 (97)	2 (3)	0	0	35 (52)	28 (41)	5 (7)	1
ASA	41	19 (66)	5 (17)	5 (17)	12	22 (56)	11 (28)	6 (15)	2
SS	15	13 (93)	1 (7)	0	1	9 (60)	6 (40)	0	0
MPNST	24	21 (88)	3 (13)	0	0	12 (50)	9 (38)	3 (13)	0
MFH	36	25 (69)	6 (17)	5 (14)	0	8 (22)	16 (44)	12 (33)	0
CCS	13	0	0	0	13	1 (8)	10 (77)	2 (15)	0
GIST	133	75 (100)	0	0	58	103 (81)	23 (18)	1 (1)	6
**Total**	**484**	358 (92)	20 (5.2)	10 (2.6)	96	325 (70)	112 (24)	29 (6)	18
		**negative: 378 (97.4)**		**positive: 10 (2.6)**		**negative: 437 (94)**		**positive: 29 (6)**	

ALT, atypical lipomatous tumor; ASA, angiosarcoma; CCS, clear cell sarcoma; DDLS, dedifferentiated liposarcoma; GIST, gastrointestinal stromal tumor; LMS, leiomyosarcoma; MFH, undifferentiated pleomorphic sarcoma; MLS, myxoid liposarcomas; MPNST, malignant; PLS, pleomorphic liposarcoma; SS, synovial sarcoma; n, number of total cases.

* immunoscore 3+ was not observed in sarcomas

*HGF*: Positive HGF immunostains were observed in 29 tumors (6.0%) (Table 1, Fig. 1). All positive samples showed moderate staining intensity in ≥10% of tumor cells (IHC score 2+). HGF overexpression was seen in undifferentiated pleomorphic sarcomas (12/36, 33%), angiosarcomas (6/39, 15%), clear cell sarcomas (2/13, 15%), malignant peripheral nerve sheath tumors (3/24, 13%), leiomyosarcomas (5/68, 7%) and GIST (1/126, 0.8%). No positive cases were present in the subgroups of atypical lipomatous tumors, dedifferentiated liposarcomas, myxoid liposarcomas, pleomorphic liposarcomas and synovial sarcomas. The FNCLCC histological grading of sarcomas correlated significantly with HGF expression (score 0–2), since higher histologic grade was associated with stronger HGF expression (p<0.001, Chi Square). 9% of tumors with FNCLCC grade 3, 7% of grade 2 and none of grade 1 were HGF immunopositive. Tumor entities which are characterized by complex karyotypes were significantly more often HGF positive than entities with specific chromosomal aberrations (p<0.001, Chi Square and Fisher’s exact). 15% of those tumors with complex karyotype and only 1% of tumors with specific aberrations were HGF immunopositive. For angiosarcomas, there was no correlation between HGF expression and pathogenesis (sporadic vs. radiation induced; p = 0.306) nor with *MYC* amplification (p = 0.478). The majority of the conventional tissue slides prepared from angiosarcomas and clear cell sarcomas were homogeneously stained with the same immunoscore in all malignant cells.

### Correlation between MET and HGF expression

388 tumors were evaluable for MET and HGF immunostains. Out of the 10 MET positive tumors, HGF results were available for 9 samples. MET and HGF immunoscores were highly significantly correlated (p<0.001, Chi Square; Table 2). 6/9 tumors (67%) were simultaneously positive for MET and HGF, i.e., 3/5 undifferentiated pleomorphic sarcomas, and 3/4 angiosarcomas showed 2+ staining for both proteins (p<0.001, Chi Square and Fisher’s exact). All three MET positive and HGF negative tumors showed weak staining intensity for HGF (score 1+).

**Table 2:** FISH results of *MET* positive cases.

	Tumor type	Cells with ≥4 *MET* signals (%)	Cells with ≥5 *MET* signals (%)	Cells with ≥15 *MET* signals (%)	*MET*/CEN7 Ratio	Average *MET* signals per cell	Amplification level (FISH score)
1	LMS	45	21.7	0	1.3	3.3	low
2	ASA	60	38.3	0	1.6	4.1	low
3	ASA	45	20	0	1.3	3.4	low
4	ASA	55	35	0	1.4	3.9	low
5	ASA	45	38.3	3.3	1.4	4.3	low
6	DDLS	40	38.3	8.3	1.2	5.7	low
7	DDLS	41.7	15	0	1.1	3.3	low
8	MLS	58.3	41.7	0	1.3	4	low
9	PLS	41.7	30	1.7	1.5	3.9	low
10	UPS	41.7	16.7	0	0.9	3.3	low
11	UPS	46.7	15	0	1.2	3.3	low
12	UPS	56.7	25	0	1	4.4	low
13	UPS	63.3	51.7	1.7	1.95	5.1	intermediate
14	UPS	96.7	96.7	53.3	1.3	15.2	high
15	UPS	71.7	65	20	1.6	8.4	high

ASA, angiosarcoma; CCS, clear cell sarcoma; CEN, centromere; DDLS, dedifferentiated liposarcoma; GIST, gastrointestinal stromal tumor; ID, identification; LMS, leiomyosarcoma; MFH, undifferentiated pleomorphic sarcoma; MLS, myxoid liposarcomas; PLS, pleomorphic liposarcoma

### MET amplification and copy number gain

**Fig 2:**
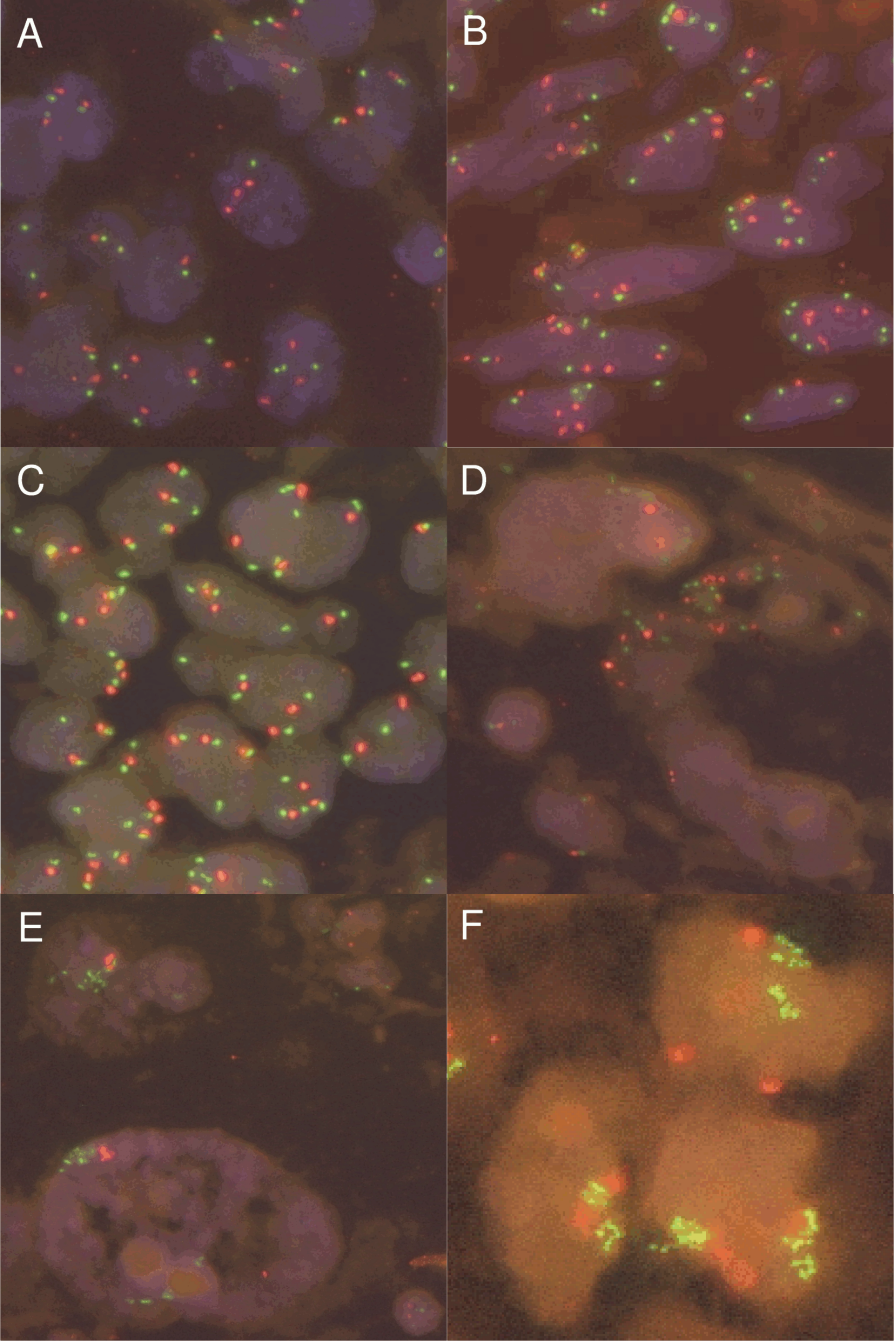
Fluorescence *in situ* hybridization for *MET*. Representative sarcomas showing different categories for *MET* gene copy number variations (see text for explanation). *MET* gene is labeled in green, centromere 7 in orange. (A) *MET* negative clear cell sarcoma, (B) *MET* negative angiosarcoma, (C) low level copy number gain in a clear cell sarcoma, (D) intermediate level copy number gain in an undifferentiated pleomorphic sarcoma, (E, F) high level amplification in undifferentiated pleomorphic sarcomas.

Fluorescence *in situ* hybridization was performed in 484 cases (Fig. 2). The FISH assay failed in 71 tumors (15%) due to absence of signals or detachment of tissue. Thus, we obtained results for 413 tumors. Fifteen tumors (4%) fulfilled the criteria for FISH positivity (low or intermediate level copy number gain or high level amplification, Table 2). High level amplification, as defined by *MET*/CEN7 ratio ≥2.0 or average *MET* gene copy number ≥6.0 or ≥15 *MET* gene copies/cell in ≥10% of tumor cells, was seen in two samples, both were undifferentiated pleomorphic sarcomas. Average *MET* gene copy number in these high level cases was 8.35 and 15.23, but ratio was below 2.0 in both cases, indicating a co-amplification of *MET* and centromeric regions of chromosome 7. Another undifferentiated pleomorphic sarcoma fulfilled the criterion for intermediate level copy number gain for *MET* (*MET* gene copy number ≥5.0/cell in ≥50% of cells) with an average *MET* gene copy number of 5.13/nucleus. *MET*/CEN7 ratio was 1.95 in this case. The other 12 positive cases were tumors with low level copy number gains (defined by ≥4.0 *MET* signals/cell in ≥40% of tumor cells). The *MET*/CEN7 ratio in this subgroup was 0.86–1.55. The percentage of tumor cells with ≥4.0 *MET* signals ranged from 41.7% to 60%. Low level copy number gain was seen in angiosarcomas (4/37, 11%), undifferentiated pleomorphic sarcomas (3/32, 9%), dedifferentiated liposarcomas (2/67, 3%), pleomorphic liposarcomas (1/10, 10%), leiomyosarcomas (1/65, 2%) and myxoid liposarcomas (1/27, 4%). Out of the 398 FISH negative tumors 194 were disomic (≤2.0 *MET* signals/cell in >90% of tumor cells) and 204 polysomic for *MET* (≥3.0 *MET* signals/cell in ≥10% of tumor cells or ≥4.0 *MET* signals/cell in <40% of tumor cells). In a subset of angiosarcomas and clear cell sarcomas whole tumor sections were hybridized all of which showed a homogeneous signal distribution. Heterogeneity, as defined by 5–50% of tumor cells fulfilling at least one of the *MET* FISH positivity criteria (modified from Vance et al. for Her2 [37]), was not observed. Subsequently to the previously described FISH analyses a subset of the undifferentiated pleomorphic sarcomas from the TMA was reevaluated on conventional tumor sections, including all six FISH positive tumors and four randomly selected FISH negative tumors. This reevaluation showed that both highly amplified tumors showed a homogeneous *MET* amplification also in the conventional sections. Furthermore, all tumors with low level gene copy number gains could be confirmed showing a homogeneous signal distribution. Only one case with intermediate level copy number gain was changed to low level gain but remained still positive. This was due to the fact that the intermediate level area was completely removed from the block by creating the TMA. Hence, all FISH positive tumors remained positive after recounting conventional tumor sections. Three of the four initially FISH negative tumors showed a heterogeneous *MET* signal distribution pattern in the larger tumor section than in the TMA cores with a focal increase in *MET* gene copy numbers resulting in focal high level amplification. Two tumors had focally more than ≥15 *MET* gene copies/cell in ≥10% of tumor cells and one tumor had a focal *MET*/CEN7 ratio ≥2.0.

The higher the FNCLCC histologic grade, the more tumors were FISH positive: 6.2% of grade 3 sarcomas (10/162; among them two high level amplified cases and one intermediate level copy number gain), 3.2% (2/63) of grade 2 and none (0/50) of grade 1 tumors were FISH positive. However, the correlation was not significant. Sarcoma entities with complex karyotypes were more often FISH positive (7.3%) than sarcomas with specific translocations or mutations (1.2%) (p = 0.001 Chi square). There was no significant correlation with etiology of angiosarcomas (spontaneous vs. radiation induced; p = 0.257) or *MYC* amplification status of angiosarcomas (p = 0.517).

### Correlation between MET amplification and MET copy number gain with MET overexpression

Results for both MET immunohistochemistry and *MET* FISH were available for 341 cases (Table 3). Notably, the two highly amplified tumors were immunonegative (scores 0 and 1+), whereas the only tumor with intermediate *MET* gene copy level reached an immunoscore 2+. Contrarily, the ten immunopositive tumors were distributed in FISH as follows: no high level, one intermediate level, one low level, and eight FISH negative tumors. 320/341 tumors (94%) were negative for both MET IHC and *MET* FISH.

**Table 3:** Correlation between *MET* amplification status and MET protein expression.

		MET immunoscore*		FISH results, n (% of available cases)				
Histological type	n		negative	low level	intermediate level	high level amplification	not available	total
atypical lipomatous tumor	43	0	33	0	0	0	9	**42 (100%)**
		1+	0	0	0	0	0	**0**
		2+	0	0	0	0	0	**0**
		n.a.	1	0	0	0	0	**1**
		**total**	**34 (100%)**	**0**	**0**	**0**	**9**	**43**
dedifferentiated liposarcoma	70	0	61	1	0	0	3	**65 (96%)**
		1+	2	1	0	0	0	**3 (4%)**
		2+	0	0	0	0	0	**0**
		n.a.	2	0	0	0	0	**2**
		**total**	**65 (97%)**	**2 (3%)**	**0**	**0**	**3**	**70**
myxoid liposarcoma	30	0	21	0	0	0	0	**21 (100%)**
		1+	0	0	0	0	0	**0**
		2+	0	0	0	0	0	**0**
		n.a.	5	1	0	0	3	**9**
		**total**	**26 (96%)**	**1 (4%)**	**0**	**0**	**3**	**30**
pleomorphic liposarcoma	10	0	9	1	0	0	0	**10 (100%)**
		1+	0	0	0	0	0	**0**
		2+	0	0	0	0	0	**0**
		n.a.	0	0	0	0	0	**0**
		**total**	**9 (90%)**	**1 (10%)**	**0**	**0**	**0**	**10**
leiomyosarcoma	69	0	62	1	0	0	4	**67 (97%)**
		1+	2	0	0	0	0	**2 (3%)**
		2+	0	0	0	0	0	**0**
		n.a.	0	0	0	0	0	**0**
		**total**	**64 (98%)**	**1 (2%)**	**0**	**0**	**4**	**69**
angiosarcoma	41	0	14	2	0	0	3	**19 (66%)**
		1+	4	0	0	0	1	**5 (17%)**
		2+	4	1	0	0	0	**5 (17%)**
		n.a.	11	1	0	0	4	**12**
		**total**	**33 (88%)**	**4 (12%)**	**0**	**0**	**4**	**41**
synovialsarcoma	15	0	11	0	0	0	2	**13 (93%)**
		1+	1	0	0	0	0	**1 (7%)**
		2+	0	0	0	0	0	**0**
		n.a.	0	0	0	0	1	**1**
		**total**	**12 (100%)**	**0**	**0**	**0**	**3**	**15**
malignant peripheral nerve sheath tumor	24	0	18	0	0	0	3	**15 (63%)**
		1+	2	0	0	0	1	**3 (13%)**
		2+	0	0	0	0	0	**0**
		n.a.	0	0	0	0	0	**0**
		**total**	**20 (100%)**	**0**	**0**	**0**	**4**	**24**
undifferentiated pleomorphic high grade sarcoma	36	0	17	3	0	1	4	**25 (69%)**
		1+	5	0	0	1	0	**6 (17%)**
		2+	4	0	1	0	0	**5 (14%)**
		n.a.	0	0	0	0	0	**0**
		**total**	**26 (81%)**	**3 (9%)**	**1 (3%)**	**2 (6%)**	**4**	**36**
clear cell sarcoma	13	0	0	0	0	0	0	0
		1+	0	0	0	0	0	0
		2+	0	0	0	0	0	0
		n.a.	11	0	0	0	2	13
		**total**	**11**	**0**	**0**	**0**	**2**	**13**
gastrointestinal stromal tumor	133	0	58	0	0	0	17	**75 (100%)**
		1+	0	0	0	0	0	**0**
		2+	0	0	0	0	0	**0**
		n.a.	40	0	0	0	18	**58**
		**total**	**98 (100%)**	**0**	0	**0**	**35**	**133**
**total**	**484**		**398 (96%)**	**12 (3%)**	**1 (0.3%)**	**2 (0.6%)**	**71**	**484**
			**negative: 398 (96%)**	**positive: 15 (4%)**				

IHC, immunohistochemistry; n, number; n.a., not available.

* immunoscore 3+ was not observed. Please note that percentages in columns or rows are based on the numbers of cases with available date not on the absolute number of samples included in that study.

## Discussion

In the evolving era of precision medicine previous studies on therapeutic targets and biomarker assays have mainly focused on large tumor entities, such as, for example, breast cancer or lung tumors. These neoplasms and the recent improvements in their therapeutic options are publicly well recognized and, thus, have led to rapid development of novel biomarker assays and enrollment of many patients in clinical trials. Contrarily, rare cancers as sarcomas and orphan diseases were not extensively studied up to now. There is, however, an urgent clinical need to improve the outcome of sarcoma patients. Roughly 5,000 estimated sarcoma related deaths per year in the U.S. indicate that the prognosis has not fundamentally changed over years. One approach to tackle this issue is to include sarcomas in prevalence studies of novel biomarkers and in early clinical trials.

In this study we have investigated the prevalence of MET alterations in a comprehensive cohort of well characterized adult-type soft tissue sarcomas and gastrointestinal stromal tumors. MET is currently studied in early and late-stage clinical trials. Activation of the MET/HGF axis can occur through diverse mechanisms suggesting several options for therapeutic intervention. Among the most promising approaches are tyrosine kinase inhibitors (i.e., small molecules) as well as anti-MET and anti-HGF antibodies. Therefore, we aimed to investigate the protein expressions of the MET kinase and its ligand HGF which might serve as biomarkers for the corresponding therapeutic antibodies. Furthermore, we investigated *MET* gene amplification which is discussed as potential predictive marker for anti-MET therapy with tyrosine kinase inhibitors.

We have included tissues from the major clinically relevant sarcoma entities which occur predominantly in adults. Based on our results we can clearly show that 94% of these tumors do not have any changes in the MET/HGF axis as measured by IHC and FISH. Notably, none of the more than 150 liposarcoma samples of any type (i.e., atypical lipomatous tumor, dedifferentiated, myxoid and pleomorphic liposarcoma) showed *MET* amplification, *MET* copy number gain or MET or HGF overexpression. This finding might be relevant for future clinical trials since liposarcomas represent one of the most common sarcoma types in adults. If not further specified “soft tissue sarcomas” are included in clinical trials with MET inhibitors it is conceivable that a considerable number of those patients would suffer from one of the liposarcoma entities which, based on our study, are unlikely to receive benefit from MET-targeted therapy. The same holds true for other clinically relevant adult-type sarcoma entities such as leiomyosarcomas and MPNST as well as for GIST.

However, we were able to show that two distinct sarcoma entities out of the eleven tumor types included in our work harbor potentially tractable MET alterations. We demonstrate that a subset of angiosarcomas and undifferentiated pleomorphic sarcomas (UPS) harbor *MET* amplifications, *MET* copy number gains or show MET/HGF overexpression. Both entities represent tumors with poor clinical outcome for which targeted therapies have not been established. Interestingly, the high level *MET* amplifications in our study are restricted to UPS where they are found in 6% of the analyzed cases. MET overexpression was found in 17% of angiosarcomas and 14% of UPS. Notably, MET overexpression—measured by IHC—was not restricted to *MET* FISH positive cases whereas MET and HGF overexpression were found to be significantly correlated. Therefore, MET overexpression in FISH negative but HGF-positive cases could indicate autocrine or paracrine stimulation of the MET/HGF axis. MET overexpression in HGF-negative tumors that are FISH negative might be due to other genetic alterations of the *MET* gene such as activating mutations or transcriptional regulation. Processing of MET-RNA, translational and protein regulation might as well result in an overexpression of MET.

The poor correlation between *MET* gene copy number gain and amplification with IHC status is peculiar. As a matter of fact, tissues collected in a phase II trial evaluating the anti-MET antibody onartuzumab in advanced NSCLC showed that *MET* amplified cases represented a subpopulation of MET positive cases as defined by IHC [38]. The lack of IHC staining in FISH positive cases in the current study could be due to technical factors; the epitope recognized by the SP44 antibody appears to be rather sensitive to tissue processing conditions and could have been deteriorated in the process of TMA generation (Hartmut Koeppen, Genentech Inc., pers. obs.). It is theoretically conceivable that the amplified copies of *MET* harbor mutations preventing transcription or translation into a stable protein which provides suitable epitopes for immunohistochemistry. It might as well be possible that the poor correlation of *MET* amplification and gene copy number alteration with IHC reflects a specific feature of sarcomas since the similar observation has recently been reported by another group [38]. It will be necessary to determine if *MET* copy number gain and amplification are frequently associated with lack of IHC staining in this disease group.

Based on our findings angiosarcomas and undifferentiated pleomorphic sarcomas rather than other frequent adult-type sarcomas should be considered for clinical trials with MET inhibitors. Angiosarcomas of the skin and the deep soft tissues occur in at least three different clinical settings: i) sporadically, ii) associated with chronic lymphedema and iii) induced by radiation. The incidence of post-radiation angiosarcomas is steadily increasing mainly since most breast cancer patients receive post-surgery radiation, and in a considerable proportion of those patients angiosarcomas occur years after treatment [39]. These lesions are barely susceptible to current treatments and, therefore, new therapeutic options are urgently needed. Interestingly, we could demonstrate that MET as well as HGF overexpression occurs in both post-radiation and primary angiosarcomas. Undifferentiated pleomorphic sarcomas represent prototypically the type of sarcomas with complex karyotypes in which numerous numerical and structural chromosomal abnormalities accumulate. From the perspective of tumor biology it is, therefore, not surprising that these neoplasms represent the only entity in which high level *MET* amplifications were found. Based on our findings we recommend applying both methodologies, *in situ* hybridization as well as immunohistochemistry, for further molecular screening procedures in prevalence studies of sarcomas. One major challenge in that context to date is that the criteria for FISH or immunopositivity are not yet consistently defined. So far as we know from previous investigations of MET in various entities it is important to adjust biomarker assays and criteria for “MET positivity” for each tumor type individually. In terms of MET immunohistochemistry we have tested various antibodies (Novocastra—clone 8F11, Invitrogen—clone 3D4, Acris—clone SP59, Santa Cruz—C-28:sc-161, data not shown) and finally decided to perform our study with the SP44 antibody which revealed the most reliable stainings in a number of test tissues. Similar findings were recently reported by Koeppen et al. [40]. They compared the staining results of different anti-MET antibodies for sensitivity and specificity and only two out of nine antibodies showed good staining results.

The immunohistochemical scoring system we worked with was four-tiered, similar to that applied for assay validation [34] and later on in clinical trials [41]. This MET scoring system consists of the evaluation of the staining intensity as well as the estimation of the proportion of stained tumor cells. Since we worked predominantly with TMA the captured tumor areas were limited, which is why we decided to reduce the threshold for the proportion of stained cells from 50% to 10%. Otherwise we attempted to stick to the previously published judgment of staining intensities. Notably, we did not observe clear-cut strong staining intensity in our sarcoma samples (as we did in positive cell line controls). However, the maximum staining score observed (score 2+ in angiosarcomas and undifferentiated pleomorphic sarcomas) was also previously considered positive in other tumor entities and it is important to recognize that our MET scoring is comparable to that in other tumors. In our study we observed 20 additional cases with 1+ MET immunostaining from various entities the clinical relevance of which remains, however, currently unclear. In some other tumor entities such as gastric carcinomas 1+ staining has previously been judged as predictively relevant positive staining.

We used immunohistochemistry also for the detection of hepatocyte growth factor overexpression, and we applied a scoring system similar to MET IHC in which the strongest staining intensity and the highest proportion of stained cells (which was observed only in non-sarcoma control tissues) was defined as 3+. We are, however, aware of some methodological limitations of this approach. To date, there are currently only few HGF antibodies on the market and none of them has been comprehensively validated for the use in clinical trials. We strongly recommend verifying our data with novel monoclonal antibodies when available or with other methods. Our preliminary data on HGF expression, however, are promising since we could demonstrate a statistically significant correlation between HGF and MET overexpression namely in angiosarcomas and undifferentiated pleomorphic sarcomas. Cases with 2+ HGF staining intensity were also seen in leiomyosarcomas, MPNST and clear cell sarcomas. These entities should, therefore, be included in confirmatory studies on HGF expression. Unfortunately we could not correlate our findings of HGF expression in clear cell sarcomas with MET immunohistochemistry due to limited tissue resources. Based on previous reports clear cell sarcomas do show MET expression to a substantial extent [22, 42, 43], thus, indicating that this tumor entity should be evaluated further for MET protein expression and *MET* copy number changes.

With more than 400 sarcoma and GIST cases included in our study this is the largest investigation on fluorescence *in situ* hybridization for the detection of *MET* amplifications in these tumors up to now. Based on our findings and on published reports we propose evaluation criteria which are easily applicable in sarcomas. Previously described criteria for *MET* FISH in different solid tumors varied, sometimes judging carcinoma cases as “positive” which were in fact far away from gene amplification. Therefore, we aimed to create a category of *MET* FISH positivity limited to truly *MET* amplified tumors. This “high level” category is defined by a *MET*/CEN7 ratio ≥2.0 or an average *MET* gene count per cell ≥6.0 or ≥15 gene copies in ≥10% of tumor cells. These criteria are generally accepted for amplification of many other genes [44, 45]. By applying these strict criteria we found that at least 6% of undifferentiated pleomorphic sarcomas belong into this category and, therefore, represent candidates for clinical trials with MET inhibitors, e.g., small molecules. Since we are aware of the fact that the clinical and therapeutical relevance of MET heterogeneity remains still to be elucidated we suggest at first to include homogeneously diffusely high level amplified tumors in screenings for clinical trials. On the other hand, we could clearly demonstrate that 96% of all sarcomas in our cohort do not harbor any significant increase in *MET* gene copy numbers (“*MET* negatives”). There is, however, a group of tumors with slightly or moderate increases in gene copy numbers which we categorized as intermediate (≥50% of tumor cells with ≥5 gene copies) or low level cases (≥40% of tumor cells with ≥4 gene copies). Currently we do not know whether these latter categories have any clinical relevance. We have, however, introduced these groupings for two reasons: i) to allow comparison of *MET* FISH data with previous results obtained by other groups since our criteria for low and intermediate level have been suggested as parameters for *MET* amplification in earlier reports on various cancers; ii) to provide a level of probability for response to anti-MET treatment. It is currently not known whether sarcoma patients will benefit from such a therapy. It would, however, be wise to include cases from that high level category with homogeneous *MET* amplification in early clinical trials at first.

Clinical trials with MET inhibitors could be discussed for sarcoma patients but will require careful planning. Based on our findings first and foremost patients with angiosarcomas or undifferentiated pleomorphic sarcomas could be considered as these entities represent sarcoma types which show first evidence of potential dysregulation of the MET pathway. Strategies to enrich for patients most likely to respond to MET-targeted therapies should be employed and should not only include IHC for MET and HGF but also determination of *MET* copy number changes. Based on preliminary data in NSCLC patients with high-level *MET* amplification seem to be most likely to benefit from MET-targeted therapy [46]. The low prevalence of sarcoma patients whose tumors may show evidence of MET pathway activation may require engagement of large clinical centers in more than one geographic area.

In summary, we investigated a comprehensive series of more than 400 adult-type sarcomas for MET and HGF overexpression as well as for *MET* gene amplification. We have established biomarker assays for immunohistochemistry and FISH and provide first data on frequency of MET alteration in sarcomas. We could demonstrate that angiosarcomas (either primary or radiation induced) and undifferentiated pleomorphic sarcomas represent those entities with a significant proportion of MET positivity. Therefore, we provide evidence that these sarcoma entities are candidates for upcoming clinical trials with compounds inhibiting the HGF/MET axis.
